# 859. An Analysis of Lipidomics in Burn Patients

**DOI:** 10.1093/jbcr/irag033.329

**Published:** 2026-04-06

**Authors:** Chloe A DiMaggio, Cara D Ramos, Dhanushka Vitharana, Amirsalar Mansouri, Abdul-Razak Masoud, Jiri Adamec, Sabyasachi Chatterjee, Jeffrey E Carter, Jonathan E Schoen, M Victoria P Miles, Alison A Smith

**Affiliations:** University Medical Center Burn Center, Louisiana State University Health Sciences Center, New Orleans, LA; Louisiana State University Health Sciences Center, New Orleans, LA; University Medical Center Burn Center, Louisiana State University Health Sciences Center, New Orleans, LA; Louisiana State University Health Sciences Center, New Orleans, LA; Louisiana State University Health Sciences Center, New Orleans, LA; Louisiana State University Health Sciences Center, New Orleans, LA; Louisiana State University Health Sciences Center, New Orleans, LA; University Medical Center Burn Center, Louisiana State University Health Sciences Center, New Orleans, LA; University Medical Center Burn Center, Louisiana State University Health Sciences Center, New Orleans, LA; University Medical Center Burn Center, Louisiana State University Health Sciences Center, Department of Surgery, New Orleans, LA; University Medical Center Burn Center, Louisiana State University Health Sciences Center, New Orleans, LA

## Abstract

**Introduction:**

Patients who sustain major burn injuries experience a pathological stress response marked by a hypermetabolic state. This change in metabolism can disrupt normal plasma lipid profiles and lead to alterations in certain biochemical processes requiring lipids. The goal of this study was to gain a better understanding of the underlying lipid metabolism dysregulation among burn patients so that we might identify potential targets for therapeutic intervention. It is hypothesized that plasma from burn patients will demonstrate significantly altered lipidome compared with healthy controls.

**Methods:**

Plasma samples were collected from 21 burn patients and matched with 16 healthy control-group participants using DUO plasma separation cards. Targeted lipidomics analysis was performed using an Agilent 1290 Infinity II LC system coupled with Agilent 6495 LC/TQ mass spectrometer following methyl-tert-butyl ether (MTBE) extraction. Lipid profiles were processed with an in-house R script, and statistical analysis was performed using the limma package (*p*<.05).

**Results:**

Ultimately, among the 770 identified lipids, 82 species from 10 different main classes were found to be dysregulated in patients with prior burn injuries. Burn patients’ plasma samples demonstrated significant increases in concentration of glycerolipids and sphingolipids while also demonstrating significant decreases in the concentration of glycerophospholipids.

**Conclusions:**

These results demonstrate that patients with prior burn injuries have altered plasma lipid profiles. Increases in both glycerolipids and sphingolipids and simultaneous decreases in glycerophospholipids point towards several metabolic differences. Primarily, these variations in lipid concentrations suggest that there is dysregulation in processes such as lipolysis, inflammatory signaling, and insulin resistance. Future studies are needed to further characterize this novel finding and determine how it can be used to improve burn care.

**Applicability of Research to Practice:**

This research can be used in practice to aid the identification of biomarkers for metabolic stress based on lipid shifts, and to advance personalized treatment approaches in burn care.

**Funding for the study:**

N/A.

